# Simple sugar supplementation abrogates exercise-induced increase in hepcidin in young men

**DOI:** 10.1186/s12970-017-0169-8

**Published:** 2017-04-20

**Authors:** Maja Tomczyk, Jakub Kortas, Damian Flis, Wojciech Skrobot, Rafal Camilleri, Jedrzej Antosiewicz

**Affiliations:** 1 Department of Biochemistry, Gdansk University of Physical Education and Sport, Kazimierza Gorskiego 1, 80-336 Gdansk, Poland; 2 Department of Recreation and Qualify Tourism, Gdansk University of PhysicalEducation and Sport, Kazimierza Górskiego 1, 80-336 Gdansk, Poland; 3 Department of Bioenergetics and Physiology of Exercise, Medical University of Gdansk, Gdansk, Poland; 4 Department of Physiotherapy, University of Physical Education and Sport, Kazimierza Gorskiego, Gdańsk, Poland

**Keywords:** Iron, Metabolism, Ferritin, CRP, Diet

## Abstract

**Background:**

At present many young people experience too much body iron accumulation. The reason of this phenomenon is not clear. There is accumulating evidences that not proper diet and lack of exercise could be a main contributing factors. This investigation assessed the effects of a diet rich in simple sugars (glucose or fructose) on exercise-induced hepcidin which is hormone regulating iron metabolism.

**Methods:**

A group of physically active young men completed an incremental exercise test before and after a 3-day diet supplemented with fructose (4 g/kg BM) or glucose (4 g/kg BM). After a 1-week break, they crossed over to the alternate mode for the subsequent 3-days period. Venous blood samples were collected before and after 1 h exercise and were analysed for serum hepcidin, IL-6, CRP, iron, and ferritin. The physiological response to exercise was also determined.

**Results:**

The concentration of hepcidin increased 1 h after exercise for the baseline test (*p* < 0.05), whereas no changes in hepcidin were observed in men whose diet was supplemented with fructose or glucose. Blood IL-6 increased significantly after exercise only in subjects supplemented with fructose. Changes in hepcidin did not correlate with shifts in serum IL-6.

**Conclusions:**

These data suggest that protective effects of exercise on excess iron accumulation in human body which is mediated by hepcidin can be abrogated by high sugar consumption which is typical for contemporary people.

## Background

Iron plays a key role in most of processes in the human body, including respiration, DNA biosynthesis, regulation of gene expression and many others. Metabolically active iron is a component of enzymes, including enzymes involved in DNA synthesis, mitochondrial and detoxifying enzymes and many more [1]. This could be the reason why many people think that if we consume or accumulate more iron is better. It is important to note that iron can be very toxic. The involvement of iron in many physiological processes is mainly related to its ability to gain and lose electrons. For example, iron in the mitochondrial respiratory chain, as a part of cytochromes, transfers electrons from NADH to oxygen. Conversely, free iron can stimulate the formation of reactive oxygen species and participate in damage to cellular structures, such as DNA, lipids or proteins [2]. Iron has been shown to mediated inflammation process induced by exercise [3]. Ferritin is a protein that stores iron and protects against its toxicity. In addition, an increase in free iron, also called the labile iron pool (LIP), induces an adaptive response, which leads to an increase in intracellular ferritin [4]. Despite this, there are several reports demonstrating that stored iron can have deleterious effects on human health and fitness [2, 5]. For example, high body iron stores were associated with low cardiovascular fitness in young men [6]. The exact mechanism of iron toxicity is not well understood, but iron bound to transferrin or ferritin does not stimulate reactive oxygen species (ROS) formation. However, non-transferrin bound iron was observed in diabetic persons [7]. In addition, experiments performed in cell culture or animals demonstrated that during stress conditions when protein kinases are activated, ferritin undergoes degradation, leading to the release of iron and increased iron-dependent ROS formation [8]. These and other studies indicate two important things: first that stored iron is not safe and second that the amount of stored iron can determine its toxicity [9]. Therefore, identifying the mechanism responsible for excess iron accumulation may be crucial for a better understanding of the pathomechanisms of iron-related morbidities.

Among factors that can influence body iron accumulation, are exercise and diet.

A diet rich in iron may certainly contribute to iron accumulation. Moreover, there are reports demonstrating that simple sugars can influence intestinal iron absorption, thereby modifying body iron stores. For example, animals on diets supplemented with fructose accumulate more iron in the liver. Because the diet of modern people is reach in sugar, mainly due to the high consumption of soft drinks and added sugar in many foods, it is important to know its effects on iron metabolism.

Conversely, exercise may cause a decrease in body iron stores. For example, decrease in body iron stores was observed in elderly women after 32 weeks of Nordic walking training [10]. By contrast, physical inactivity, which has been recognized as epidemic in modern societies, may also have an impact on iron metabolism [11, 12]. Regular exercise reduces body iron stores; therefore, the lack of exercise may be responsible for body iron accumulation [10, 13]. The effect of exercise on iron metabolism may be mediated by an increase in the hepcidin concentration [14]. Hepcidin is a 25 aa peptide that inhibits intestinal iron absorption or its release from storage tissues, such as the liver and other organs [15]. The purpose of this study was to re-examine the role of simple sugar in a diet on exercise induced changes in iron metabolism.

Any factor that modifies the effects of exercise on blood hepcidin may contribute to impaired iron metabolism. In our study, we hypothesized that a diet rich in simple sugars (glucose and fructose) may modify the effect of exercise on blood hepcidin.

Furthermore, blood ferritin is a good marker of body iron stores if inflammation is not present.

## Methods

### Subjects

The study involved 17 healthy, physically active but not highly trained, college-aged men (Table 1). The study protocol used a randomised, crossover, blind design. Study subjects performed exercise test after 3 days of control diet and then were randomised to either 3 days of diet supplemented with fructose (4 g/kg BM) or glucose (4 g/kg BM), and then after a 1-week break, crossed over to the alternate mode for the subsequent 3-day period. During the 1-week break participants maintained they daily routines and diet was not control. The participants were fully informed and gave written informed consent to participate in this study as approved by the Medical Research Ethics Committee of the Medical University of Gdansk in Poland. Details that might disclose the identity of the subjects under study have been omitted.

**Table 1:** Anthropometric and physiological characteristics of participants (*n* = 17)

Variable	X	SD	Me	V	Confidence interval	
					−95%	+95%
Age [years]	21,12	1,11	21	5,26	20,55	21,69
Weight [kg]	77,6	5,56	77,3	7,17	74,74	80,46
Height [cm]	183	5,36	183	2,93	180,24	185,76
Fat [%]	12,21	3,34	10,8	27,4	10,49	13,92
FFM [kg]	68,19	6,28	69,2	9,21	64,97	71,42
TBW [kg]	49,95	4,59	50,6	9,2	47,59	52,31
VO_2max_ [mL · kg^−1^ · min^−1^]	51,06	8,9	49	17,43	46,48	55,63

*Abbreviations*: *X* means, *SD* standard deviation, *Me* median, *V* coefficient of variation, *BMI* Body Mass Index, *Fat* fat mass, *Fat %* percentage of body fat, *FFM* free fat mass, *TBW* total body water, *VO2max* maximal oxygen uptake expressed in relatively values, *Pmax* maximal power

### Diet

The nutritional components of the diet are presented in Table 1 Participants ate 5 meals a day at set times over a period of 3 days. The respondents declared that they would not eat other meals than those that fall within the scope of the study. The daily value of the diet was 3541.90 kcal (14,819.66 kJ) (Table 2). One hour before the start of the test, all participants received a breakfast with energy 613 kcal (2565 kJ). The nutritional value of the meals was estimated using the dietary calculator Nutritionist 2012 (Software Jumar 2012), based on the nutritional value determined by Polish Institute Food and Nutrition.

**Table 2:** The nutritional and energy value of 3-day diet (without drinks)

Energy value (kcal)	3541.90
Carbohydrates (g)	589.9
Proteins (g)	156.3
Fat (g)	72.2
Fibre (g)	30.3

### Aerobic test

**Fig. 1:**
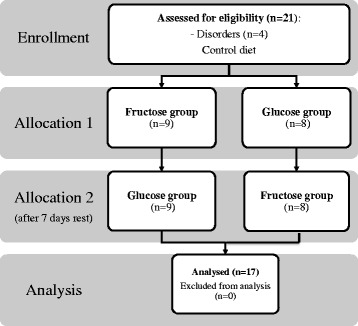
Schematic presentation of the experimental design

The aerobic test was performed at the beginning of the study after 3 days of diet which was the same for all of the participants. Then after 3 days of sugar supplementation. The study design is presented in Fig. 1. Maximal oxygen uptake (VO2 max) was determined using a cycle ergometry test on an electromagnetically braked cycle ergometer (ER 900 Jaeger, Germany/Viasys Health Care). Participants were allowed a 5-min warm-up period at an intensity of 1.5 W kg with a pedalling cadence of 60 rpm. Immediately after the warm-up, the participants began VO2 max testing by cycling at increasingly difficult workloads, in which resistance was increased by 25 W min until the participant reached the point of volitional exhaustion. Breath-by-breath pulmonary gas exchange was measured (Oxycon-Pro, Jaeger-Viasys Health Care, Hochberg, Germany) throughout the VO2 max test. The O_2_ and CO_2_ analysers were calibrated before each test using standard gases of known concentrations in accordance with the manufacturer’s guidelines.

### Blood analysis

A professional nurse collected blood samples. Samples were obtained from an antecubital vein into single-use containers with an anticoagulant (EDTA K2) before and 1 h after the aerobic test. The serum hepcidin levels were determined using a DRG ELISA kit CRP, IL6.

## Results

Of the 21 subjects who were enrolled in the study, four were excluded because of failure to complete the second exercise test (Fig. 1). Therefore, only 17 subjects are included in the analyses. No difference was observed between fructose vs glucose supplementation in the dietary intake for total kilocalories, total grams of protein, or percentage protein. As discussed it the methods, they remained on the same diet for 3 days before the exercise test.

### Effect of fructose and glucose supplementation on blood hepcidin

**Fig. 2:**
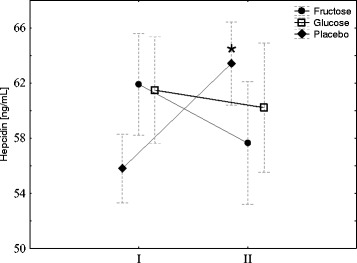
Fructose or glucose supplementation abrogates exercise-induced hepcidin. The average level of IL-6 in young men before (*I*) and 1 h after aerobic test (*II*) by group of supplementation. Tags represent average values, frames - standard deviations, * - statistically significant differences between (*I*) and (*II*), *p* < 0,05

To evaluate the effect of fructose or glucose supplementation on iron metabolism, the subjects underwent an exercise test to exhaustion and the blood hepcidin was then measured. As shown in Fig. 2, exercise induced a significant increase in blood hepcidin in subjects who were on the diet without supplementation (placebo). Conversely, no changes in hepcidin concentration were observed in subjects whose diet was supplemented with fructose or glucose. Notably, 3 days of diet supplemented with fructose or glucose did not influence the resting blood hepcidin.

**Fig. 3:**
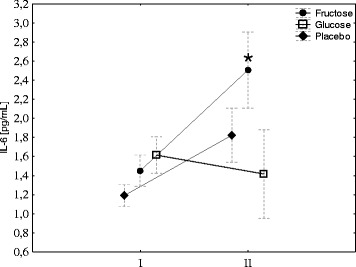
Fructose supplementation augments exercise-induced IL-6. The average level of IL-6 in young men before (*I*) and 1 h after aerobic test (*II*) by group of supplementation. Tags represent average values, frames - standard deviations, * - statistically significant differences between (*I*) and (*II*), *p* < 0,05

**Fig. 4:**
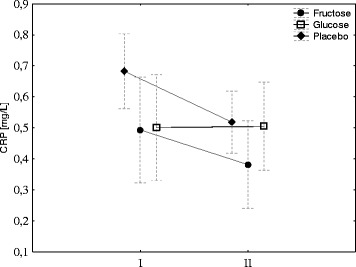
Fructose or glucose supplementation has no effect on CRP. The average level of CRP in young men before (*I*) and 1 h after aerobic test (*II*) by group of supplementation. Tags represent average values, frames - standard deviations

Hepcidin biosynthesis is stimulated by IL-6; therefore, its concentration was evaluated before and after exercise. Exercise induced a significant increase in IL-6 in the fructose group and had no effects in the G group. A small increase in IL-6 was observed in the placebo group; however, the changes were not significant (Fig. 3). Neither exercise nor diet had effect on blood CRP concentration (Fig. 4).

## Discussion

In the present study, we demonstrated that a diet rich in simple sugars, fructose or glucose modulates iron metabolism in young men. Sugar can stimulate intestinal iron absorption; however, the mechanism of this has not been fully elucidated [16]. It is proposed that fructose increases dietary non-heme iron absorption, possibly by chelating and/or reducing iron into the ferrous form [17].

Many scientific papers state that iron deficiency is the most common nutrition deficit in the world. Conversely, in the Western world, iron overload is much more common than iron deficiency. For example, a study performed on elderly subjects demonstrated that 12.9% of the studied population were iron overloaded (serum ferritin (SF) >300 μg/L in men and 200 μg/L in women), whereas only 3% were iron deficient (SF < 12 μg/L) [18].

A study performed on young men demonstrated that most had blood ferritin concentrations above 150 μg/L and this was associated with low cardiovascular fitness [6]. The authors estimated that in the USA alone, approximately 24.8 M young men could have ferritin above this value. Notably, ferritin the reference value is 16 to 300 μg/L; however, an increasing number of studies have demonstrated that persons with ferritin above 100 μg/L are at risk for several morbidities, including cancer diabetes, heart attack and others [19–21]. Therefore, it is important to determine the reason for excess iron accumulation in so many people. Certainly, diet and exercise are two important determinants of iron status. Recently, we demonstrated that regular training reduced body iron stores in the elderly [10]. Moreover, highly trained young men are characterised by lower body iron stores compared to untrained young men [14]. Why regular training reduces body iron stores is not precisely understood; however, an increase in blood hepcidin may be an important cause. A single exercise session can lead to an increase in blood hepcidin, [14, 22] which can lead to lower intestinal iron absorption because it blocks ferroportin, a protein that exports iron from enterocytes into blood [15].

High sugar consumption is typical for highly developed countries. The average sugar consumption in the United States is approximately 70 kg per person per year [23]. A diet rich in sugar may increase physical performance by stimulating glycogen biosynthesis. This is one reason why many athletes consume sugar-rich diets. If super compensation of glycogen is to be achieved, the diet should contain approximately 70% carbohydrates. Unfortunately, often sweets or other products rich in sucrose or simple sugars are an essential part of the athlete diet. This is why this study diet was supplemented with fructose or glucose. Doses of 4 g/kg body weight per day were applied. Here, we present evidence that supplementation of the diet with fructose or glucose abrogates the increase in blood hepcidin after exercise. It was previously reported that not all subjects respond the same way; therefore, they were divided into responders and nonresponders, those whose blood hepcidin increased or did not change after exercise, respectively. The reasons for this are not known. Peeling and co-workers suggested that it is due to the difference in body iron stores, and persons with low iron stores do not respond to exercise by increasing hepcidin biosynthesis. Consistent with this, blood hepcidin did not change after 100 km in runners who were also characterized by relatively low ferritin levels (53 μg/L) [24]. The results of this study indicate that the difference could be a result of the diet.

Exercise stimulates IL-6 release from skeletal muscle and induces hepcidin biosynthesis in the liver [25]. We observed that exercise significantly increased the IL-6 concentration only in subjects whose diet was supplemented with fructose. If it is, consider that IL-6 is a skeletal muscle origin [26] these data suggest that fructose has some effect on muscle metabolism distinct than glucose. In addition it rather excludes the role of IL-6 in inducing an increase in hepcidin after exercise and are consistent our previous studies on ultra-marathoners in whom an increase in IL-6 was not accompanied by changes in blood hepcidin [24].

## Conclusion

In conclusion, this study demonstrated that high consumption of fructose or glucose abrogates the exercise-induced increase in blood hepcidin. These data suggest that high sugar is a reason for excess body iron accumulation.

## Abbreviations

BM: Body mass
LIP: Labile iron pool
ROS: Reactive oxygen species
SF: Serum ferritin
VO2: Max maximal oxygen uptake
